# Exploring the potential of digital biomarkers as a measure of brain health ‘capital’

**DOI:** 10.1038/s41746-025-01675-2

**Published:** 2025-06-05

**Authors:** Dylan Powell, Stephanie A. Adams, Donncha Mullin, Miles Welstead, John E. Harrison, Craig Ritchie

**Affiliations:** 1 Faculty of Health Sciences & Sport, University of Stirling, Stirling, UK; 2 Moray House School of Education and Sport, University of Edinburgh, Edinburgh, UK; 3 UK Collaborating Centre on Injury and Illness Prevention in Sport (UKCCIIS), Edinburgh, UK; 4 Scottish Brain Sciences, Edinburgh, UK; 5 Division of Psychiatry, Centre for Clinical Brain Sciences, University of Edinburgh, Edinburgh, UK; 6 Alzheimer Scotland Dementia Research Centre, University of Edinburgh, Edinburgh, UK; 7 Alzheimercentrum Amsterdam, AUMC, Amsterdam, The Netherlands; 8 Centre for Affective Disorders, IOPPN, KCL, London, UK; 9 School of Medicine, University of St Andrews, St Andrews, UK

**Keywords:** Neurology, Public health

## Abstract

Neurological conditions, including dementia, pose a major public health challenge, contributing to a significant and growing clinical, economic, and societal burden. Traditionally, research and clinical practice have focused on diseases like dementia in isolation. However, in an ageing, multimorbid population, this approach is becoming increasingly inadequate. Recognising brain health as a lifelong attribute influenced by various health determinants, this paper explores the concept of brain health, identifies key challenges in assessing it effectively, and examines how digital biomarkers could provide a versatile measurement framework to enhance monitoring and facilitate earlier intervention. Finally, we outline future directions to help advance definitions of meaningful aspects of brain health integration, and practical adoption of digital biomarkers, enhancing our capacity to measure and preserve ‘brain health capital’ or ‘brain span’ across the lifecourse.

## Introduction

Neurological conditions such as dementia are among the most pressing public health challenges of the 21^st^ century, with significant clinical, economic, and societal impacts^1^. They are now the leading cause of disability and the second leading cause of death worldwide^2^, underscoring the urgent need for improved prevention, targeted treatment, and personalised care. Traditionally, research, practice, and policy has focussed on specific diseases such as dementia and stroke. However, in an ageing multi-morbid population, this single-disease approach has proven inadequate, increasing the need for timely, comprehensive brain health assessments to support earlier, more effective interventions and optimise ‘brain health capital’ across the lifespan. Despite advancements in our understanding of shared disease processes, often beginning long before symptoms appear, measurement approaches remain reactive, disease-specific, remain fragmented and reactive, often relying on subjective self-reports that fail to capture the complexity and long-term trajectory of cognitive decline^3^. A clear illustration of discrepancy between self-report and objective performance was the observations of typical control study participants 48-hours after the administration of the antipsychotic drug, haloperidol. Study participants reported being restored to usual levels of attention but were markedly impaired on reaction time measures of attention^4^. This finding exemplifies the limitations inherent to subjective self-assessments and underscores the need for more objective, real-time cognitive assessments^5^.

## Brain health as lifelong asset

Recognising the potential for multi-purpose interventions, the concept of **brain health** has emerged, challenging siloed research and shifting focus towards proactive, objective, scalable, and longitudinal assessment strategies. In 2022, the World Health Organisation (WHO) defined brain health as “the state of brain functioning across cognitive, sensory, social-emotional, behavioural, and motor domains, allowing a person to realize their full potential over the life course, irrespective of the presence or absence of disorders.” This expanded definition positions brain health as a fundamental form of capital-‘brain health capital’ and acknowledges the broader influences of neurological conditions, injury, or lifestyle factors on brain functioning, highlighting significant consequences for both individual well-being and societal outcomes^6–8^. This paper explores the concept of brain health, identifies key challenges in assessing it effectively, and examines how digital biomarkers could provide a versatile measurement framework to enhance monitoring and facilitate early intervention. Advances in digital health tools offer new opportunities for earlier detection, personalized interventions, and a more nuanced understanding of brain function. By leveraging these innovations, we can move toward a proactive, scalable, and continuous approach to brain health assessment-one that addresses this global health priority^4^.

## Opportunities afforded by digital biomarkers

The future of brain health measurement lies in the integration of different multimodal datasets, allowing for a more holistic understanding of their interactions over time. The concept of brain health as a lifelong journey encourages researchers and clinicians to move beyond short-term assessments and consider long-term influences on cognitive and neurological function. This requires a paradigm shift-one where brain health is seen as a complex, interconnected system, where interventions targeting one domain (such as physical activity or stress management) may yield positive cascading effects in others (such as cognitive function and emotional well-being).

Digital biomarkers are well positioned to help facilitate this shift, providing scalable, objective, and continuous monitoring tools capable of capturing subtle physiological and behavioural changes^9^. Below, we outline the opportunity that digital health technologies and associated digital biomarkers may help address in traditional limitations to help optimise brain health capital across the lifespan.

## Towards scalable disease agnostic measures

A fundamental limitation in current brain health assessments is the lack of widely used, scalable, disease-agnostic tools capable of evaluating cognitive, motor, emotional, and sensory functions simultaneously. Traditional methods are typically condition-specific, targeting isolated neurological diseases such as Alzheimer’s or Parkinson’s, and failing to capture the broader, lifelong trajectory of brain health. This siloed approach restricts our understanding and proactive management of brain health overlooking the dynamic and multidimensional interactions across different functional domains.

For example, cognitive assessments commonly employed in clinical trials for Alzheimer’s disease, including the Mini-Mental State Examination (MMSE) and the Alzheimer’s Disease Assessment Scale–Cognitive Subscale (ADAS-cog), predominantly focus on episodic memory. However, these can lack content validity and are restricted largely to episodic memory assessment, failing to measure executive function, attention, or other critical cognitive domains^10^. This can lead to more fragmented evaluation or siloed analysis, where different domains of brain function are considered separately, overlooking any potential interaction^11^. Additionally, current tools often lack sufficient sensitivity to detect subtle, pre-symptomatic changes, particularly in younger or healthy individuals^3^. The widespread use of wearables and mobile devices has provided an opportunity to augment by collecting real-world data across multiple functional domains as a proxy for brain health capital^1^.

### Moving away from Subjective Self-Reported Approaches

Many current brain health assessments rely heavily on subjective self-reporting and clinician-administered tests, which can introduce significant variability and bias. Factors such as mood, motivation, and environmental conditions can influence results, leading to inconsistencies in cognitive test outcomes. For example, performance on widely used assessments like the Montreal Cognitive Assessment (MoCA) can be affected by factors such as anxiety, fatigue, or recent sleep quality, reducing the reliability of these measures^12,13^. These subjective, time-bound methods may be less sensitive in measuring gradual or long-term changes in brain function, which could be crucial for early detection of cognitive decline. Thus, preserving brain health capital requires objective, continuous, real-time monitoring methods, such as digital biomarkers, to complement traditional approaches and provide reliable, longitudinal insights into brain function^7^.

### Objective assessment across the life course

While advanced neuroimaging techniques such as MRI, PET scans, and EEGs provide detailed insights into brain structure and activity, they can be cost-prohibitive, resource-intensive, and not always practical for routine monitoring across populations. These imaging methods typically provide only single, time-bound “snapshots,” making it challenging to detect subtle or early-stage changes crucial for proactive brain health management. In response, there is increasing interest in more portable, affordable, and accessible approaches, such as digital health technologies and sensors. These technologies offer the potential for continuous, real-world monitoring, capturing more subtle and dynamic shifts in brain function that might otherwise not be measured by existing approaches^8^.

## Future directions and recommendations

**Fig. 1:**
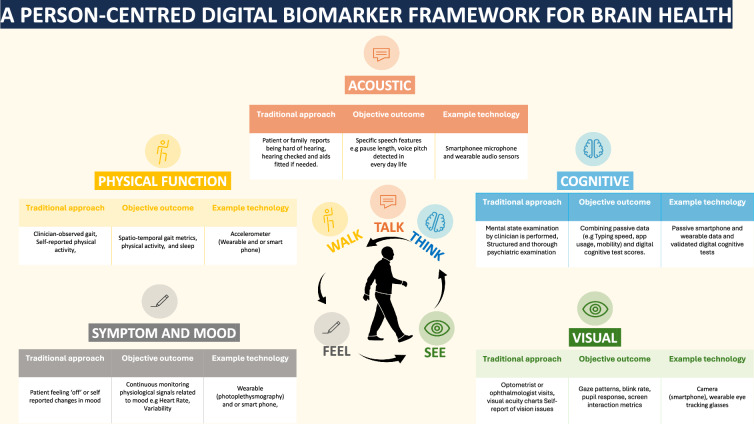
A person-centred framework for brain health.

The opportunity of digital measurements has opened up new avenues for comprehensively brain health detection of subtle changes that may aid in diagnosis and prognosis. However, to fully realise these benefits, there are a number of difficult challenges to navigate (Fig. 1).

### Integration of multi-modal data and real-world evidence

**Fig. 2:**
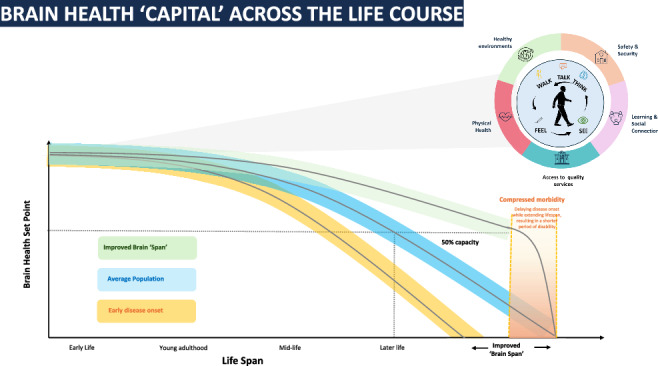
Brain Health across the Life course.

Brain health is shaped by both intrinsic factors (e.g., neuroplasticity, pruning) and extrinsic influences (e.g., physical comorbidities, environmental conditions, healthcare access). As outlined in Fig. 2, these factors may collectively inform an individual’s *brain health capital*-here referred to as a cumulative resource reflecting one’s evolving cognitive and functional capacity. By integrating multi-modal data from clinical diagnostics, wearable sensors, and mobile health platforms, there is now an opportunity to construct a more comprehensive and dynamic picture of brain health. Importantly, the use of real-world evidence-captured through continuous, passive monitoring, allows for the detection of subtle day-to-day changes that may precede overt clinical symptoms. This shift from episodic assessment to continuous, personalised monitoring can enable earlier intervention, support proactive care strategies, and ultimately enhance our ability to measure, preserve, and promote brain health capital across diverse populations, as outlined in the WHO position paper, Optimizing brain health across the life course^14^.

### Validation and contextualization

For digital biomarkers to have clinical utility, rigorous standards must be met, including reliability, validity, and sensitivity. A critical requirement for scalable digital biomarkers is comprehensive content validity, ensuring accurate assessment across multiple cognitive domains, including episodic and semantic memory, attention, executive function, and emotional and sensory domains. Future research must prioritise robust validation studies, aiming to standardise and benchmark digital biomarkers against existing gold-standard clinical measures, thus ensuring these novel methods are both scientifically sound and clinically meaningful to patients^15^.

### Ethical and regulatory considerations

Using digital tools may act as a more holistic for brain health, offering passive, non-invasive ways to track and intervene in brain health continuously. However, shifting toward widespread, long-term continuous monitoring of brain health raises significant concerns around personal data usage, including issues related to trust, privacy, ethics, and acceptability among users^16^. Addressing concerns through transparent data governance, user control mechanisms, and Patient and Public Involvement (PPI) may help to navigate these challenges, ensuring innovations are both effective and acceptable to end users. Developing user-centred data control mechanisms and ensuring that digital innovations align with ethical and regulatory standards will be crucial for the successful adoption and acceptance of these technologies as a meaningful measures of brain health in clinical practice and public health settings^17^.

## Conclusion

Brain health is a multidimensional and lifelong concept that encompasses more than the absence of disease or injury. With growing recognition of the many factors influencing brain function, from lifestyle choices to social determinants, a more holistic view and thus a mechanism to measure brain health is needed. Traditional assessment methods, which often rely on episodic, condition-specific, and reactive approaches, are increasingly being recognised as insufficient for capturing the full trajectory of brain health across the lifespan. The integration of digital biomarkers and emerging technologies presents a transformative opportunity to help address these challenges. By expanding the focus beyond specific injuries or diseases, we can move towards a more integrated, comprehensive understanding of brain health that considers how different factors interact and impact overall human health. This opens up new avenues for engagement, education, and interventions designed to promote lifelong brain health and preserved cognitive ‘capital’, ultimately improving both individual outcomes and public health on a global scale.

## Data Availability

No datasets were generated or analysed during the current study.
